# Oncolytic adenoviruses and immunopeptidomics: a convenient marriage

**DOI:** 10.1002/1878-0261.13648

**Published:** 2024-04-01

**Authors:** Marc Garcia‐Moure, Andrew G. Gillard, Marta M. Alonso, Juan Fueyo, Candelaria Gomez‐Manzano

**Affiliations:** ^1^ Department of Neuro‐Oncology The University of Texas MD Anderson Cancer Center Houston TX USA; ^2^ Department of Pediatrics Clinica Universidad de Navarra Pamplona Spain; ^3^ Program of Solid Tumors Foundation for the Applied Medical Research Pamplona Spain

**Keywords:** adenovirus, cancer, mesothelioma, personalized, vaccine, virotherapy

## Abstract

Oncolytic viruses (OVs) are biological therapeutic agents that selectively destroy cancer cells while sparing normal healthy cells. Besides direct oncolysis, OV infection induces a proinflammatory shift in the tumor microenvironment and the release of tumor‐associated antigens (TAAs) that might induce an anti‐tumor immunity. Due to their immunostimulatory effect, OVs have been explored for cancer vaccination against specific TAAs. However, this approach usually requires genetic modification of the virus and the production of a new viral vector for each target, which is difficult to implement for low prevalent antigens. In a recent study, Chiaro et al. presented an elegant proof of concept on how to implement the PeptiCRAd vaccination platform to overcome this limitation for the treatment of mesothelioma. Authors showed the feasibility of identifying immunogenic TAAs in human mesothelioma and using them to coat oncolytic adenovirus particles. The result was a customized virus‐based cancer vaccine that circumvents time and resource‐consuming steps incurred from genetically engineering viruses. Although some questions remain to be addressed, this interesting approach suggests novel strategies for personalized cancer medicine using oncolytic virotherapy.

## Graphical abstract

This commentary focuses on the personalized development pipeline of therapeutic cancer vaccines (PeptiCRAd) for the treatment of mesothelioma. The patient tumor immunopeptidome is subject to *in‐silico* software predictions to identify candidate peptides, followed by validation of immunogenicity using patient‐derived immune cells *in vitro*. Validated peptides are then bound to oncolytic adenovirus to form PeptiCRAd, which can be administered intratumorally.

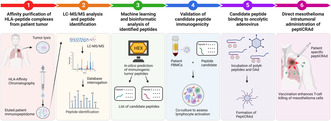

**Abbreviations**
DAMP: damage‐associated molecular patterns
MHC‐I: major histocompatibility complex class I
OV: oncolytic virus
PAMP: pathogen‐associated molecular patterns
PBMC: peripheral blood mononuclear cells
TAA: tumor‐associated antigen
TME: tumor microenvironment

The interest in oncolytic virotherapy has been steadily increasing over the last 25 years. Oncolytic viruses (OVs) are either naturally occurring or genetically modified viruses that preferentially infect and/or replicate in cancer cells, leading to tumor cell lysis and the propagation of newly generated virions to the surrounding neighboring tumor cells [1]. The initial tenet was that the administration of oncolytic viruses would result in tumor shrinkage and complete remission of tumors following several cycles of infection‐replication‐lysis viral propagation. This scenario was the blueprint that researchers had in mind to develop a plethora of OVs. However, data from clinical trials and the use of immunocompetent models of cancer revealed that the initial oncolysis phase was followed by an anti‐tumor immune response that was required to eradicate the tumor. Therefore, the focus on OVs veered towards enhancing the immune arm of this therapy.

The administration of the virus is inevitably accompanied by the exposure of virus‐borne pathogen‐associated molecular patterns (PAMPs) that promote a proinflammatory shift within the tumor microenvironment (TME). In addition, the concomitant production of damage‐associated molecular patterns (DAMPs) and spreading of tumor‐associated antigens (TAAs) by the virus‐mediated cell tumor lysis results in the enhancement of the anti‐tumor immune response. The mounting of an effective tumor‐specific immune reaction may lead to long‐lasting anti‐tumor responses, abscopal effect in distant lesions, and the development of an anti‐tumor immune memory. In summary, OVs are now considered a legitimate class of immunotherapy [2].

Despite observing clinical responses in several phase I studies using OVs for the treatment of cancer, including extended survival in a subset of patients [3, 4, 5], there is still room for improvement. Several aspects should be addressed to enhance the efficacy of viroimmunotherapy. While the inflammation triggered by the viral infection is required for the anti‐tumor immune response, the injection of OVs is inevitably accompanied by the generation of virus‐specific immune responses that can eventually lead to a premature clearance of the pathogen and a weak anti‐tumor response due to the immunodominance of viral epitopes. Therefore, redirecting the immune reaction toward the tumor is a crucial aspect of recent research in virotherapy. Moreover, it is important to invigorate the anti‐tumor immune response even further to overcome the highly immunosuppressive TME that might limit the efficacy of OVs.

In a recent manuscript by Chiaro et al. [6], the authors studied the synergistic effect of immunopeptidomics‐identified peptides in combination with viral immunotherapy using the PeptiCRAd vaccine platform. PeptiCRAd is a strategy developed by the authors consisting of an oncolytic adenovirus (OAd) coated with tumor‐specific peptides. This combines viral immunogenicity with the cancer specificity of the peptides, generating a “cancer vaccine” [7]. The efficacy of PeptiCRAd *in vivo* has been successfully tested in various tumor types. In the recently published manuscript, the investigators specifically propose an approach to improve the efficacy of virotherapy for mesothelioma based on implementing the PeptiCRAd strategy to an oncolytic chimeric adenovirus Ad5/3 expressing the T cell and DC activators OX40L and CD40L, respectively. Once administered, the virus contributes to the production of inflammation in TME, while tumor peptides carried by the virus are presented to their specific T cells to potentiate an anti‐tumor response. The idea of using adenoviruses for vaccination has been widely explored for cancer [8] and infectious diseases [9], but in most cases, it involves the genetic modification of the virus. After the adenovirus infection, the antigen will be expressed until the virus is completely cleared. However, the need to engineer and manufacture a new adenovirus for each target antigen is a resource and time‐consuming process that constitutes an important hurdle for the clinical implementation of genetically modified viruses as vaccines. Therefore, although genetic engineering of viruses for vaccination is a feasible option for highly prevalent targets, their application for less frequent antigens is very limited.

In this matter, the PeptiCRAd approach represents an advantage in the context of personalized therapy in cancer. As the authors show here and in previous work, using the PeptiCRAd technology, a generic adenovirus particle can be easily modified to incorporate any given peptide target by electrostatic interactions. The authors also provide a proof of concept demonstrating the feasibility of identifying potential immunogenic tumor antigens among the MHC‐I immunopeptidome of the tumor and loading them into the adenovirus for clinical use (Fig. 1). Therefore, a single batch of adenovirus will serve as a carrier for personalized targets, saving time and resources required to produce new viruses.

**Fig. 1:**
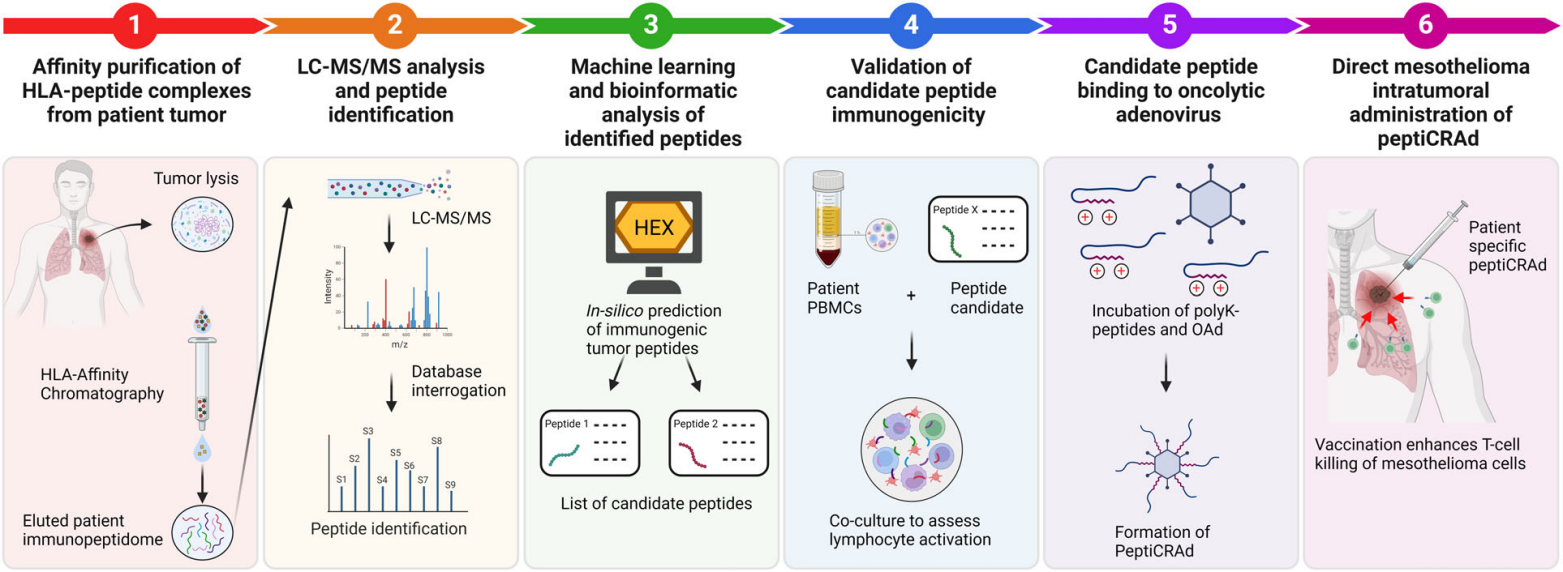
Graphical abstract: Proposed PeptiCRAd pipeline for treatment of mesothelioma. (1) Major histocompatibility complex (MHC) I peptides are immunopurified from the surface of patient tumor cells. (2) The peptides are analyzed by liquid chromatography with tandem mass spectrometry (LC–MS/MS). (3) The generated list of peptides is interrogated with hex software to expedite candidate peptide selection. (4) The selected peptides then go through a functional characterization for their immunogenicity profile and are validated *in vitro* using patient‐derived peripheral blood mononuclear cells (PBMCs). (5) The best candidates are poly‐lysine‐modified and bound to the oncolytic adenovirus (OAd) surface. (6) The resulting therapeutic cancer vaccine (PeptiCRAd) can be injected into mesothelioma intratumorally [Created with BioRender.com].

This study delves into an innovative method designed to strengthen the immune response directed specifically at tumors during cancer virotherapy. Overall, the authors demonstrate that the use of immunopeptidomic analysis in combination with oncolytic immunotherapy represents a feasible and effective strategy to tackle untreatable tumors and may propel vertical progress in the fields of cancer virotherapy and cancer immunotherapy. The work encompasses comprehensive *in vitro* and *in vivo* studies conducted on various mouse and human cell lines, as well as human tissue specimens. As with any thorough study, this report prompts further questions.

PeptiCRAd vaccination works as a first‐shot strategy. The coating of viruses with peptides precludes their persistence after the initial infection. Alternative strategies, such as cloning of the peptides within the virus genome, would, however, allow the expression of the antigens in every round of replication, multiplying the number of cells expressing them. The authors have extensively validated the capacity of PeptiCRAd to instigate anti‐tumor responses in immunocompetent mice for various cancer models, obtaining encouraging results. However, replication of human adenoviruses is limited in murine cells [10], and the expression of viral genes, including late genes, is severely impaired in these cells. The lack of viral replication is a substantial limitation of the proposed approach and may impact the outcome PeptiCRAd system in preclinical studies. We cannot discard the idea that if the virus replicates with more efficiency, such as in the human setting, the amplification and persistence of viral antigens could eventually result in the immunodominance of the anti‐viral response, thus significantly attenuating the anti‐tumor response.

The success of PeptiCRAd will also be influenced by the selection of the antigen targets. In this study, the authors screened the immunopeptidome in different mesothelioma models and patient samples. More than 80% of the eluted peptides were haplotype‐specific, and more than 60% were found in healthy tissues. Although these findings underscore the relevance of a versatile system such as PeptiCRAd over genetically modified viruses, they also highlight a need to develop robust bioinformatic algorithms to identify the most appropriate tumor peptides for personalized vaccination. The bioinformatic curation of the candidate peptides used by the authors relies on their affinity for MHC‐I and an *in‐silico* prediction of their immunogenicity based on their sequence similarity with previously reported peptides. Then, they generate a filtered list of the most relevant candidates to test their immunogenicity in PBMCs. However, there are other important factors to be considered during the initial screening, such as the haplotype of the patient, the tumor specificity, the driving role of the antigen, or their clonality, among others. For example, a high clonality of tumor antigens is crucial. Otherwise, there is a high risk of selecting low clonal antigens, thus resulting in the destruction of a small subset of tumor cells. The implementation of artificial intelligence and machine learning technologies will probably provide a substantial improvement in integrating all this information to provide a relevant list of potential candidates.

In summary, PeptiCRAd is an exciting platform for developing personalized virus‐based vaccination for cancer. At the same time, it opens avenues for interesting improvements in the area of viroimmunotherapy. Therefore, the recently published manuscript by Chiaro and colleagues represents a further step towards implementing oncolytic virotherapy in the clinical arena.

## Author contributions

Conceptualization, MG‐M, CG‐M and JF; literature analysis, MG‐M, CG‐M, JF and AGG; writing‐original draft preparation, MG‐M; review and editing, MG‐M, AGG, MMA, JF and CG‐M. All authors have read and agreed to the published version of the manuscript.

## Conflict of interest

The authors declare no conflict of interest.
